# Interface engineering for gain perovskite photodetectors with extremely high external quantum efficiency†

**DOI:** 10.1039/d0ra06618d

**Published:** 2020-09-04

**Authors:** Xinyu Zhao, Lixiang Huang, Yukun Wang, Xinglin Zhu, Lei Li, Guoxin Li, Wenhong Sun

**Affiliations:** Research Center for Optoelectronic Materials and Devices, School of Physical Science and Technology, Guangxi University Nanning 530004 China ykwang0929@163.com 20180001@gxu.edu.cn; Key Laboratory of New Processing Technology for Nonferrous Metals and Materials (Ministry of Education), Guangxi University Nanning 530004 China

## Abstract

Efficient CH_3_NH_3_PbI_3_ photodetectors (PDs) with an extremely high gain of the maximum external quantum efficiency (EQE) of 140 000% within the ultraviolet region to the near infrared region (NIR) and an extremely high responsivity (*R*) under a low bias of −5 V were successfully fabricated. The fabricated devices manifested outstanding environmental stability with only 10% degradation of EQE after being exposed to air for 24 h. These obtained results indicate the promising potential of perovskite PDs for visible light communication applications.

## Abstract 2

Efficient CH_3_NH_3_PbI_3_ photodetectors (PDs) with an extremely high gain of the maximum external quantum efficiency (EQE) of 140 000% within the ultraviolet region to the near infrared region (NIR) and an extremely high responsivity (*R*) under a low bias of −5 V were successfully fabricated.

## Graphical abstract

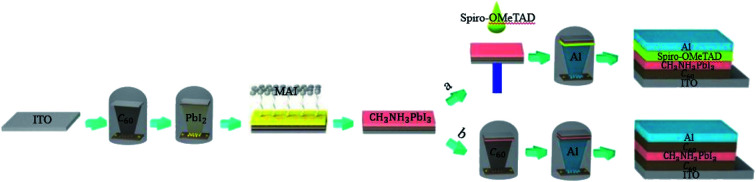

## 1. Introduction

Organic/inorganic hybrid lead halide perovskite solar cells have aroused significant interest in the photovoltaic research community due to their excellent optoelectronic properties including intense broadband absorption, tunable band gaps, high carrier mobility and long carrier diffusion lengths.^1–5^ In the last decade, the power conversion efficiency of the perovskite solar cells has remarkably increased from 3.8% to over 25.2% at a rapid rate; hence, they can compete with the commercial silicon solar cells provided their stability can be continuously improved.^2,6–19^ It is due to the exceptional semiconducting properties that organic/inorganic hybrid perovskites have also been utilized to fabricate ultrafast and highly sensitive photodetectors.^20,21^ Yang *et al.* fabricated a novel solution-processed perovskite photodetector in 2014.^22^ Operating at room temperature, the photodetector exhibited a large detectivity of 10^14^ jones, a linear dynamic range of 100 decibels, and a fast photoresponse with a bandwidth of 3 MHz. Meredith *et al.* optimized the thickness of the double fullerene transport interlayer and achieved an external quantum efficiency (EQE) of 80% at 400–760 nm, measured-limited LDR of 170 dB, and a detectivity over 10^12^ jones.^23^ Huang *et al.* reported narrow band photodetection using hybrid perovskite single-crystal-based photodetectors (PDs).^24^ In these aforesaid researches, photodiode-type PDs have been used. In this type of PDs, a single absorbed photon can trigger one or less electron flow, thus resulting in an EQE of less than 100%, which will weaken the light sensing in a variety of applications such as industries, defense and scientific research. Therefore, it is indispensable to increase the number of charges flowing through an external circuit per incident photon (defined as the photoconductive gain).^25^

Gain is essential for highly sensitive devices for allowing the extraction of a higher number of charge carriers for each absorbed photon. In general, the trap states are beneficial to achieve gain. In the trap states, minority charge carriers are captured by traps, thus allowing the majority carriers to flow through the external circuit. In perovskite PDs, gain can be obtained due to the trap states. Huang *et al.*, used the intrinsic hole trap states of perovskite thin films to obtain a maximum gain of 495 at a very low driving voltage of −1 V.^26^ Ruan *et al.* used F4TCNQ as electron traps for generating hole gain and obtained an EQE of 60 000%.^27^ Similarly, traps are often used to produce high gain in organic gain PDs, traps are often used to produce high gain.^28^ Therefore, an effective hole or electron trap is an imminent requirement to realize high gain of perovskite PDs.

In the present study, gain CH_3_NH_3_PbI_3_ PDs were successfully fabricated. High-quality perovskite thin films were synthesized on C_60_*via* vapor-assisted and vacuum-processed techniques. The C_60_-modified indium tin oxide (ITO) substrate improved the crystal quality of the CH_3_NH_3_PbI_3_ thin films and significantly increased the EQE of the PDs. Furthermore, due to the tunneling effect between C_60_ and ITO, the incorporation of the C_60_ layer between CH_3_NH_3_PbI_3_ thin films and top-electrode enhanced the photocurrent of CH_3_NH_3_PbI_3_ PDs. Benefitting from the tunneling effect, efficient CH_3_NH_3_PbI_3_ PDs with an extremely high gain with the maximum EQE of 140 000% within the ultraviolet region to the near infrared region (NIR) and an extremely high responsivity (*R*) under a low bias of −5 V were fabricated. The fabricated devices manifested outstanding environmental stability with only 10% degradation of EQE after being exposed to air for 24 h. These obtained results indicate the promising potential of perovskite PDs for visible light communication applications.

## 2. Experimental details

### 2.1 Materials

Lead iodide (PbI_2_), 4-*tert*-butylpyridine (*t*BP), and bis(trifluoromethylsulfonyl)imide lithium salt (Li-TFSI) were purchased from Xi'an Polymer Light Technology Corp. Spiro-OMeTAD and C_60_ were purchased from Sigma-Aldrich.

#### Synthesis of CH_3_NH_3_I

CH_3_NH_3_I was synthesized through the reaction of 24 mL methylamine (33 wt% in ethanol, Aldrich) and 10 mL hydroiodic acid (57 wt% in water, Aladdin, China) in 100 mL ethanol under ice bath for 2 h with stirring. The precipitate was collected with a rotary evaporator at 50 °C to remove the solvent. Then, the product was recrystallized from ethanol. The crystals were filtered and washed three times with diethyl ether.^12^ Finally, the solid was dried overnight at 60 °C in a vacuum oven. All the materials were used as received without further purification.

### 2.2 Fabrication of perovskite solar cells and gain photodetectors

**Fig. 1:**
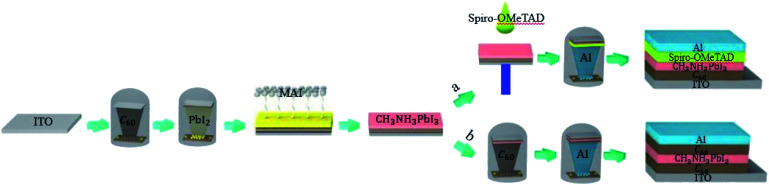
Schematic representation of the experimental process.

Indium tin oxide (ITO) was sequentially cleaned in deionized water, acetone, and ethanol under ultrasonication for 20 min each and then treated with oxygen plasma for 15 min. C_60_ was evaporated on ITO at a rate of 0.05 nm s^−1^, PbI_2_ was then evaporated on C_60_-coated ITO, and subsequently, the substrates were transported into a N_2_ glove box. CH_3_NH_3_I powder was spread out on the PbI_2_-coated substrates and heated at 160 °C for 4.5 h to fabricate the perovskite thin films. After depositing the perovskite layer, spiro-OMeTAD was spin-coated on the substrates (Route a in Fig. 1) or C_60_ was evaporated on perovskite thin films (Route b in Fig. 1). Finally, 100 nm Al was deposited as the back contact *via* thermal evaporation. The schematic of the experimental process is presented in Fig. 1.

### 2.3 Device characterization

X-ray diffraction (XRD) patterns were recorded on a XRD system under monochromatic Cu Kα irradiation. Photovoltaic performances were measured on a Keithley 2400 source meter at AM 1.5 G illumination (100 mW cm^−2^). The dark current voltage characteristics were analyzed by a Keithley 236 source measuring unit. A setup made by the Beijing 7-star Optical Instruments Co. Ltd was used to measure the quantum efficiency.

## 3. Results and discussion

**Fig. 2:**
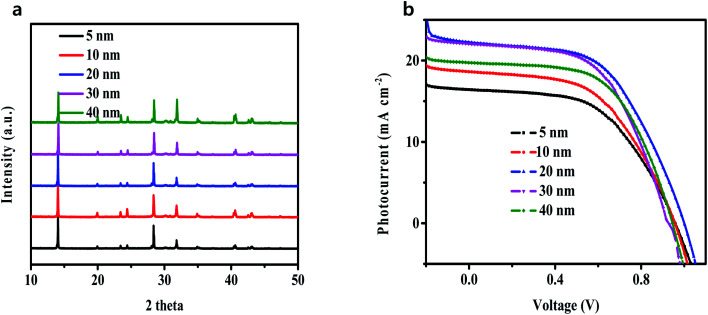
(a) Normalized XRD of perovskite films on different thicknesses of the C_60_ layer; (b) the corresponding photocurrent density–voltage curves of devices with different thicknesses of the C_60_ layer.

The performance of perovskite devices greatly depends on the crystallinity and grain size of the perovskite thin films. To obtain the coverage and uniformity of the perovskite thin films, PbI_2_ thin films were first evaporated on ITO glass substrates coated with a C_60_ layer and then annealed in CH_3_NH_3_I vapor at 160 °C in N_2_ atmosphere for 4.5 h. In order to investigate the existence of perovskite thin films, XRD on the CH_3_NH_3_PbI_3_ films was performed. Fig. 2a and S1† display the normalized and absolute XRD patterns of the CH_3_NH_3_PbI_3_ films with different thicknesses of the C_60_ layer. All the films exhibit similar diffraction peaks located at 14.2° and 28.4°, which are assigned to the (110) and (220) planes, respectively. Fig. S2a–e† present the scanning electron microscopy (SEM) images of these perovskite films. The small difference in XRD and SEM indicates that CH_3_NH_3_PbI_3_ films remain stable at high temperature and possess an orthorhombic crystal structure with high crystallinity, agreeing well with the previous reports.^29^

To directly evaluate the performance of the as-prepared high-quality perovskite layers on the ITO/C_60_ substrate, a series of solar cells with layered inverted structure of glass/ITO/C_60_/perovskite/spiro-OMeTAD/Al was fabricated (Route a in Fig. 1), where ITO was the cathode, C_60_ was the electron transporting layer, CH_3_NH_3_PbI_3_ was the active layer, spiro-OMeTAD served as the hole transporting layer (HTL) and aluminum (Al) acted as the anode. As the thickness of C_60_ can greatly influence the performance of the solar cells, perovskite solar cells with different C_60_ thicknesses (marked as 0 nm, 5 nm, 10 nm, 20 nm, 30 nm and 40 nm) were fabricated, and their corresponding current density–voltage curves are summarized in Fig. 2b and S3,† which were measured under simulated AM 1.5 G solar irradiation in a N_2_ atmosphere. It is noticeable from Fig. S1† that the cells without C_60_ manifested an extremely poor performance. This is because the direct contact between the perovskite thin films and the ITO electrode resulted in a large number of carrier interfacial recombination. Although some researchers had directly grown efficient planar lead halide perovskite devices on ITO or FTO substrates,^21,30^ those perovskites were prepared based on a low-temperature solution method. In the present study, the perovskite was processed on a very high-temperature substrate; hence, the direct contact with the substrate concentrated the heat on the perovskite, causing the interior of the perovskite to be destroyed. After a 5 nm thick C_60_ film was deposited between the ITO and the perovskite, the heat was shared by C_60_; thus, the perovskite could keep the lattice stable, and the cell performance was significantly improved due to the efficient extraction and transmission of electron, as shown in Fig. 2b. With the increase in the C_60_ thickness, the PCE of the fabricated perovskite solar cells first increased and then decreased perhaps due to the following reasons: (i) when the thickness of C_60_ was less than 20 nm, C_60_ had a strong ability to prevent hole injection from the ITO electrode and occupied the main position; therefore, the PCE of the cells was improved; (ii) when the thickness of C_60_ was more than 20 nm, C_60_ had a strong ability to absorb the incident photons; thus, less light was absorbed by the perovskite active layer, and consequently, the PCE of the cells was reduced.

**Fig. 3:**
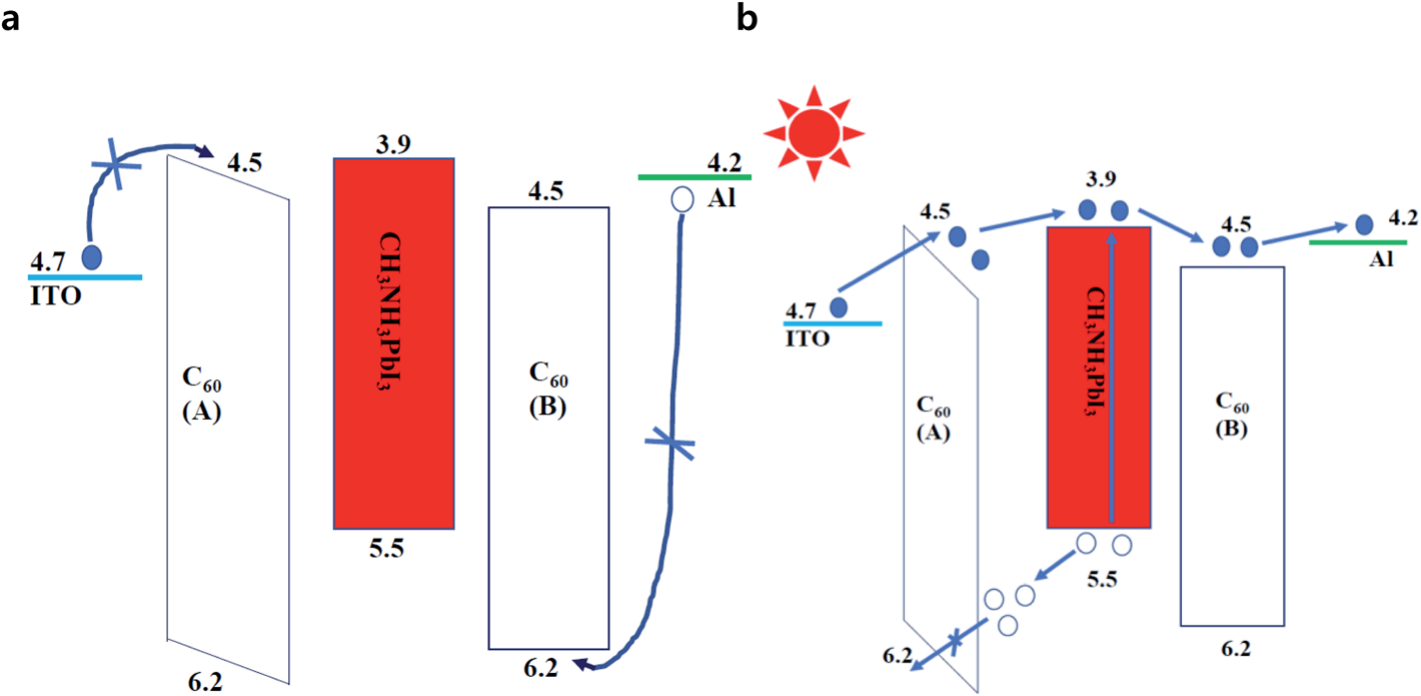
Schematic energy level diagram of the fabricated gain perovskite PDs under dark and light conditions. The hollow and solid circles represented holes and electrons, respectively. (a) Under dark conditions, it was not easy to inject electrons and holes into the device because of barrier potential; (b) under light conditions, the barrier width of C_60_ (A) layer was narrowed and a large number of electrons easily tunneled from ITO to C_60_.

**Fig. 4:**
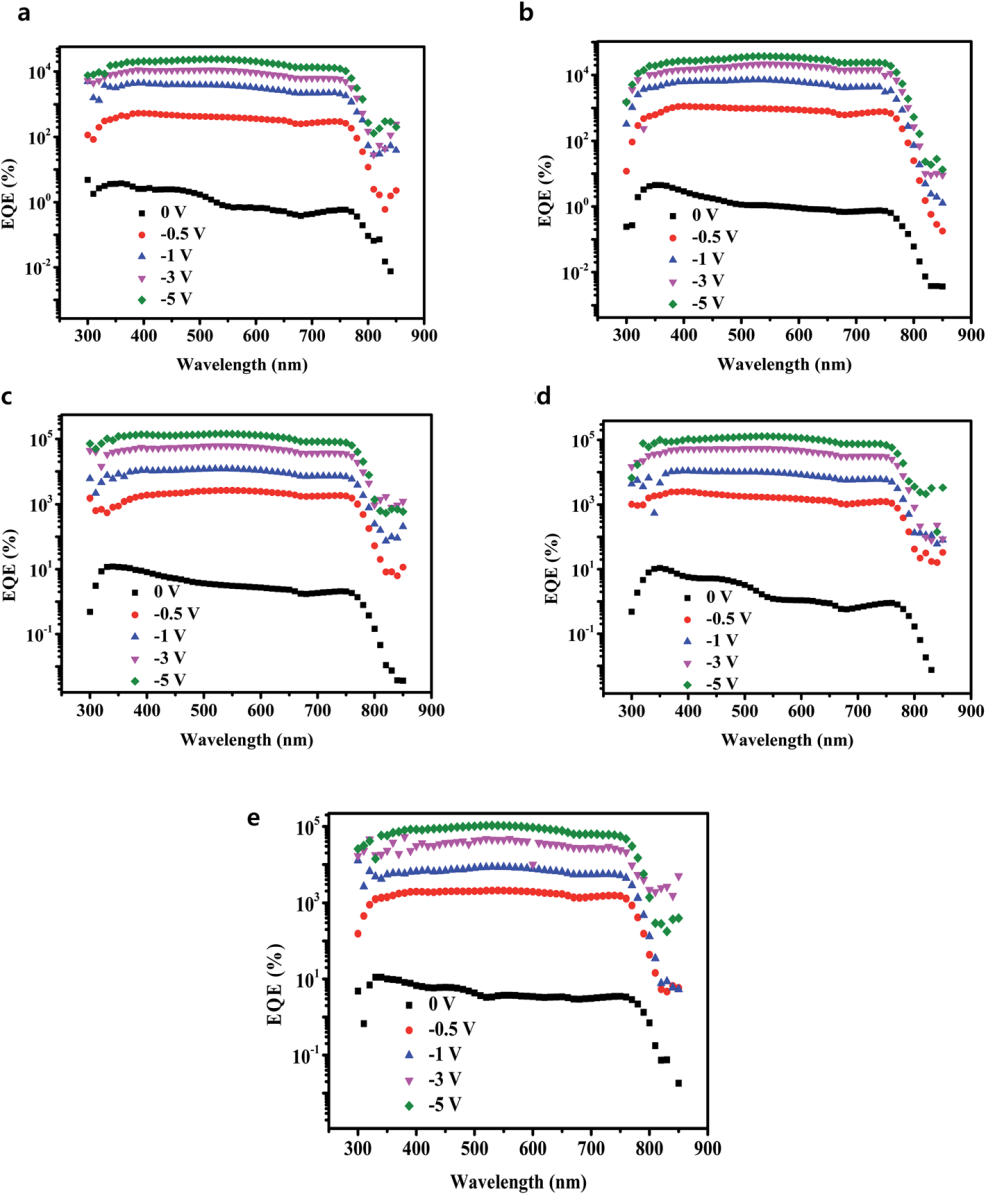
EQE spectrum *vs.* wavelength of gain PDs with different thicknesses of C_60_ (A) layer at different voltages. (a) 5 nm; (b) 10 nm; (c) 20 nm; (d) 30 nm; (e) 40 nm.

The as-prepared perovskite thin films were used to fabricate a gain perovskite photodetector with a configuration of ITO/C_60_ (A)/CH_3_NH_3_PbI_3_/C_60_ (B)/Al (Route b in Fig. 1), and the corresponding energy level diagram of the device is illustrated in Fig. 3a. Under dark conditions, it was not easy to inject electrons and holes into the device because of the barrier potential between the C_60_ layer and the electrodes. Under light conditions and a negative external bias voltage, the photons were absorbed by the CH_3_NH_3_PbI_3_ layer and the excitons generated by photons were easily separated at room temperature. After electric field drove the electrons to transport through the perovskite layer and C_60_ (B), they were collected by the Al anode, and at the same time, the holes moved to the interface between C_60_ (A) and CH_3_NH_3_PbI_3_. Due to the deep energy level (traps for holes) and low hole mobility of C_60_, the holes were trapped in the interface. After the accumulation of a large number of holes, they created a considerable voltage drop in the C_60_ (A) layer, narrowing the barrier width and bending the energy band of C_60_ (A). Hence, a large number of electrons easily tunneled from ITO to C_60_ and flowed through the device. The simulation diagram of this process is exhibited in Fig. 3b. To reveal the validity of our analysis, the EQE spectra of photodetectors with different C_60_ thicknesses at different reverse biases were recorded, as shown in Fig. 4a–e. It is discernible that EQE was extremely low at low voltages, which should be the reason that the accumulated holes were very few and the tunneling effect was not obvious. However, when the voltage was over −0.5 V, the EQE values of all the PDs exceeded 1000%. With increasing voltages, the EQE values continuously increased and exceed 30 000% at −5 V. The device with 20 nm C_60_ (A) manifested the best performance. When the voltage reached −5 V, the EQE was over 140 000%. These results are far greater than the values for photodiode PDs,^22^ even better than many other gain perovskite PDs.^26,27^

**Fig. 5:**
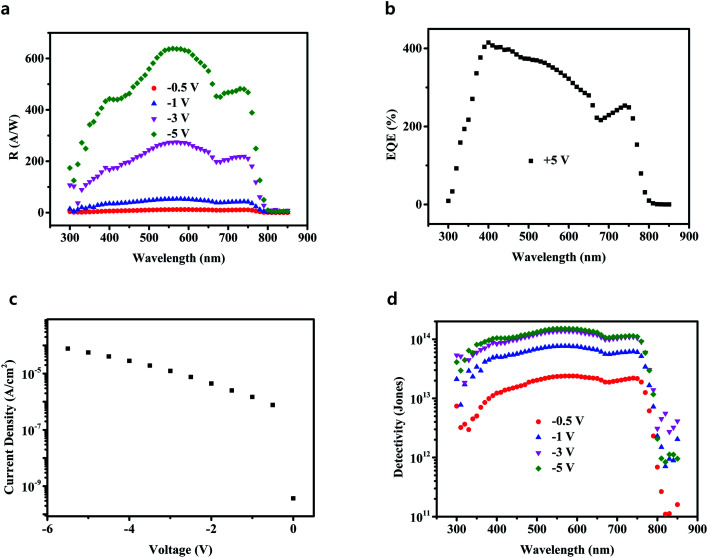
(a) *R* spectra *vs.* wavelength measured under different bias conditions; (b) EQE *vs.* wavelength under a forward bias voltage of +5 V; (c) dark current density *vs.* voltage; (d) D* spectra *vs.* wavelength measured under different bias conditions.

Obviously, a number of electrons tunneling injection resulted from the hole accumulation and the higher voltage resulted in the higher EQE. In order to simplify the illustration, in the following stage we focused on gain perovskite PDs with the best performance having 20 nm thick C_60_ (A) layer. Having obtained the EQE of the gain perovskite PDs, the responsivity (*R*) could be calculated carefully. *R* is an important metrics for the PDs, which is a function of EQE and indicates how efficiently photodetectors respond to incident optical signals. The result of *R* at different voltages is summarized in Fig. 5a, and the maximum *R* was over 600 A W^−1^. For photodiode PDs, *R* is generally less than 1 A W^−1^,^12^ whereas the investigated gain PDs had much larger values.

In the gain PDs, C_60_ layers were used twice; however, each time, they had different functions. C_60_ (A) served as the hole blocking layer to accumulate the holes. Because of the process of the preparation of perovskite thin films, a small amount of PbI_2_ was present at the bottom of the perovskite thin films, leading to the optimization of the perovskite active layers.^31^ It was very difficult to recombine the holes restricted at the interface; hence, a large number of holes bent the band of the C_60_ (A) layer. The C_60_ (B) layer had three different functions: (i) transport electrons from the perovskite layer because of its high electron mobility, (ii) block holes from the Al cathode due to its deep energy level and extremely low hole mobility, and (iii) passivate the perovskite surface. In fact, top of perovskite thin films was very rough. If holes are accumulated here, they would be easily recombined by traps or other dislocations. To prove our assumption, the EQE of gain PDs under a forward bias voltage of +5 V was measured, and a very poor performance was observed, as shown in Fig. 5b. Moreover, normalized EQE of PDs with different thicknesses of C_60_ (B) (@550 nm and −5 V) is shown in Fig. S4.† These results could also prove the above hypothesis. C_60_ (B) transported the electrons from the perovskite layer and the thickness of C_60_ (B) had little influence on the EQE.

The dark current density *versus* voltage curves for the gain devices was plotted in Fig. 5c. Although C_60_ had a strong ability to block the holes injection, it had a weak ability to stop electrons injected from the electrodes. Hence, the dark current density (*J*_d_) of the investigated gain PDs was one or two order higher than those of the common photodiode PDs.^22–24^ Having established EQE and *J*_d_, we warily calculated another key figure of merit of the PDs denoted as the specific detectivity (D*). In general, D* is mainly determined based on the EQE and noise current. However, due to the limited experimental conditions, D* values were obtained from EQE and *J*_d_. As had been reported, if the shot noise was derived from the dark current, D* could be expressed as the following equation:*D* = *R*/(2*eJ*_d_)^1/2^where *e* is the quantity of electric charges, *R* and *J*_d_ had been established in Fig. 5a and c, respectively. Fig. 5d showed the D* spectra of the gain perovskite PDs at different voltages. The intensities of the D* spectra increased with increasing voltage mainly due to the extremely high EQE. The phenomenon was different from the photodiode PDs.^12^

**Fig. 6:**
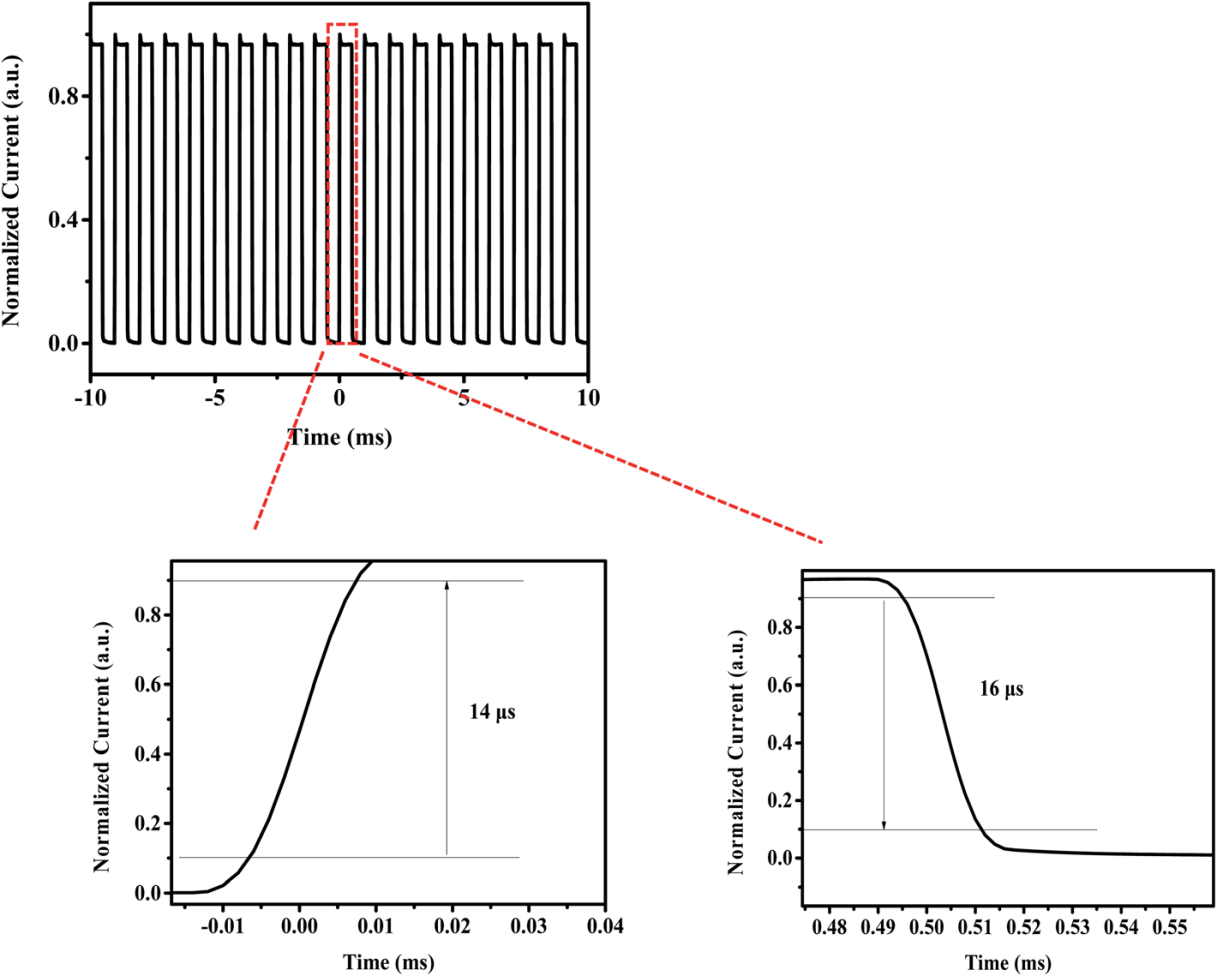
Normalized photocurrent response *vs.* time, the rise time is 14 μs and the fall time is 16 μs.

Subsequently, we measured the transient response of gain photodetectors. In general, the response time is strongly related to the charge transport and collection. In the gain PDs, holes were restricted at the interface between C_60_ and perovskites, and hence, the charge transport was hindered, and their response times were slower than those of photodiode PDs. Fig. 6 presents the results of the response time. As reported,^32^ the rise time was defined as the time required for the output signals to increase from 10% to 90% of the saturated photocurrent. Similarly, the fall time was defined as the time required for the output signals to decrease from 90% to 10% of the saturated photocurrent. In the present experiment, a rise time of 14 μs and a fall time of 16 μs were detected.

**Fig. 7:**
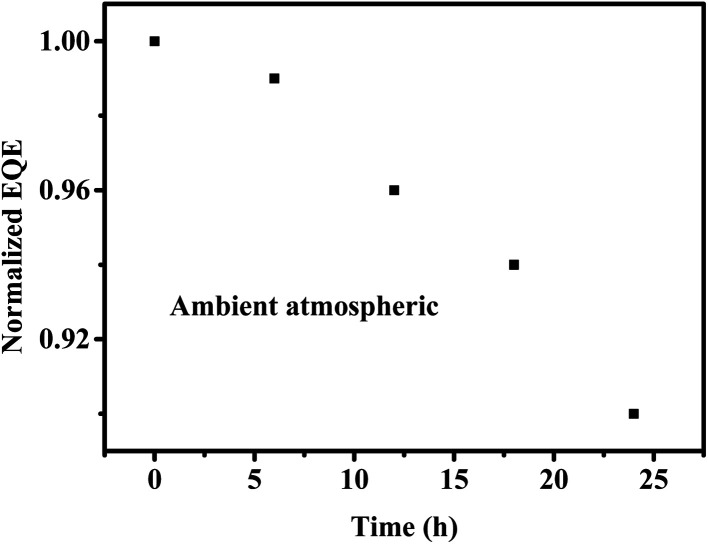
Normalized EQE (@550 nm and −5 V) *vs.* time.

In order to demonstrate the excellent stability of the gain perovskite PDs, aging tests were performed under ambient atmospheric conditions, and the corresponding results are presented in Fig. 7. It is noticeable that the gain perovskite PDs manifested great stability with less than 10% EQE loss (@550 nm and −5 V) after being exposed to air for 24 h. This large stability indicates that the gain perovskite PDs could function well under moisture and oxygen. Finally, some characteristic parameters for perovskite photodetectors in previous reports are listed in Table 1, indicating that our device is competitive in this field.

**Table 1:** Comparison of the characteristic parameters for some previous perovskite photodetectors

Material bias [V]	Responsivity (wavelength)	D* [jones]	*τ*_r_/*τ*_d_	Reference
CH_3_NH_3_PbI_3_-0.1	0.21 A W^−1^ (white light)	7.4 × 10^12^	5/5 μs	33
CH_3_NH_3_PbI_3_-0	0.29 A W^−1^ (490 nm)	3.29 × 10^12^	20/17 μs	34
CH_3_NH_3_PbI_3_-0	0.4 A W^−1^ (600 nm)	1 × 10^12^	1.2/3.2 μs	35
CH_3_NH_3_PbI_3_-1	242 A W^−1^ (740 nm)	—	10/5.7 μs	26
CH_3_NH_3_PbBr_3_-5	Over 4000 A W^−1^ (525 nm)	Over 3 × 10^13^	25/25 μs	36
CH_3_NH_3_PbI_3_-1	4.95 A W^−1^ (530 nm)	2 × 10^13^	0.1/0.1 ms	37
CH_3_NH_3_PbI_3_-4	7.56 A W^−1^ (360 nm)	—	170/220 μs	38
CH_3_NH_3_PbI_3_-2	110 A W^−1^ (532 nm)	2.2 × 10^11^	2.1/2.9 s	39
CH_3_NH_3_PbI_3_-1	260 A W^−1^ (532 nm)	8.1 × 10^14^	9/27 μs	27
CH_3_NH_3_PbI_3_-5	600 A W^−1^ (550 nm)	1 × 10^14^	14/16 μs	This work

## 4. Conclusion

In the study, efficient hybrid perovskite PDs were synthesized by growing CH_3_NH_3_PbI_3_ layer on the C_60_ surface through vapor-assisted and vacuum-processed techniques. The C_60_-modified ITO substrate improved the crystal quality of CH_3_NH_3_PbI_3_ thin films and significantly increased the EQE of the PDs. Furthermore, due to the tunneling effect between C_60_ and ITO, the incorporation of the C_60_ layer between CH_3_NH_3_PbI_3_ thin films and top-electrode enhanced the photocurrent of the CH_3_NH_3_PbI_3_ PDs. Benefitting from the tunneling effect, efficient CH_3_NH_3_PbI_3_ PDs with an extremely high gain of the maximum EQE of 140 000% from the ultraviolet range to the NIR and showing an extremely high *R* under a low bias of −5 V were fabricated. The fabricated devices manifested outstanding environmental stability with only 10% degradation of EQE after being exposed to air for 24 h. These obtained results indicate the promising potential of perovskite PDs for visible light communication applications.

## Conflicts of interest

There are no conflicts to declare.

## Supplementary Material

RA-010-D0RA06618D-s001
