# Supplementary material for: Interface engineering for gain perovskite photodetectors with extremely high external quantum efficiency
Source: RSC Adv. 2020 Sep 4;10(54):32976–83. doi: 10.1039/d0ra06618d (PMC9056631; doi:10.1039/d0ra06618d)
Supplement: RA-010-D0RA06618D-s001 [file RA-010-D0RA06618D-s001.pdf]

Electronic Supplementary Material (ESI) for RSC Advances.
This journal is © The Royal Society of Chemistry 2020

Supporting Information

Interface engineering for gain perovskite photodetectors with an extremely high external quantum efficiency

Xinyu Zhao[#1], Lixiang Huang[#1], Yukun Wang[#1]*, Xinglin Zhu[1], Lei Li[1], Guoxin Li[1], Wenhong Sun[1,2]*

[1] Research Center for Optoelectronic Materials and Devices, School of Physical Science and Technology, Guangxi University, Nanning 530004, China.

[2] Key Laboratory of New Processing Technology for Nonferrous Metals and Materials (Ministry of Education), Guangxi University, Nanning 530004, China.

*corresponding e-mail address: ykwang0929@163.com(Y.Wang); 20180001@gxu.edu.cn(W.Sun)

#These authors contributed equally

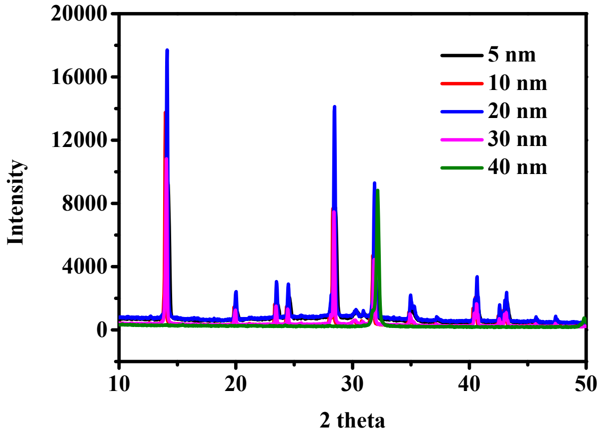

Figure S1 Absolute XRD of perovskite films on different thicknesses of C_{60} layer

a

5 nm

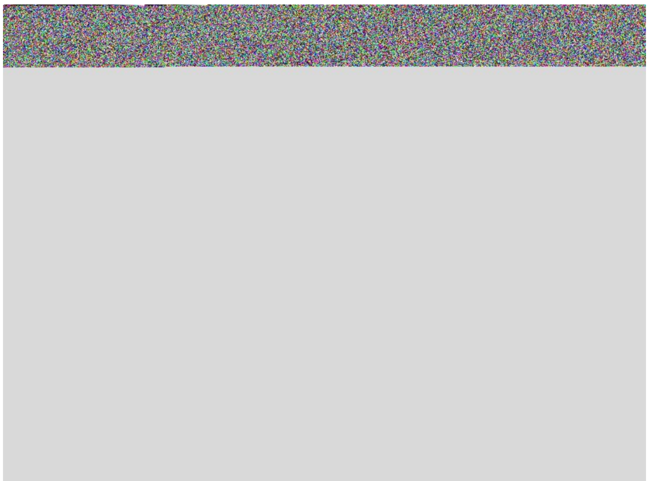

b

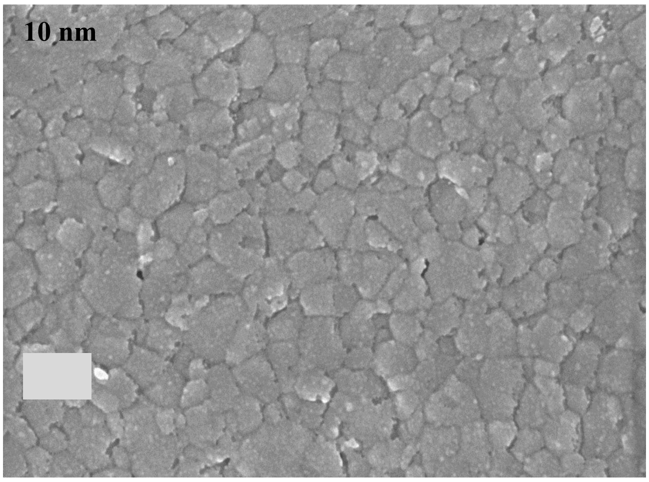

c

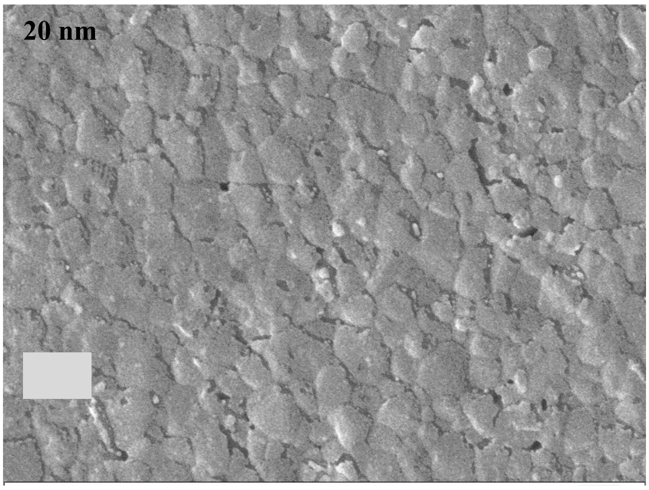

30 nm

d

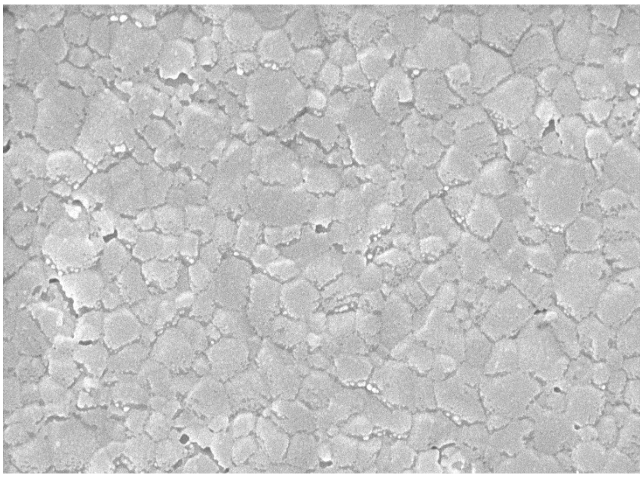

e

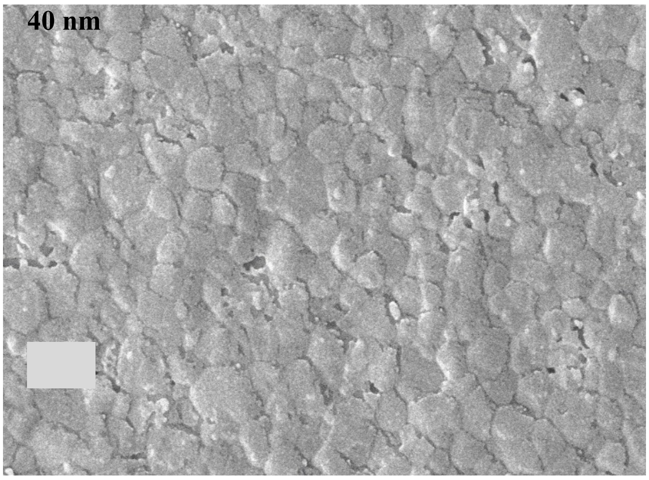

Figure S2 a-e SEM of perovskite films on different thicknesses of C_{60} layer

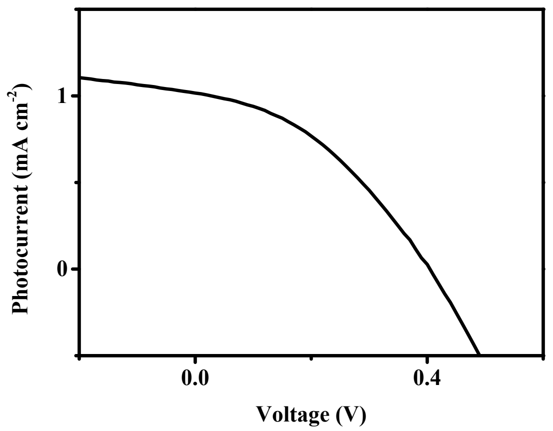

Figure S3 Current density vs. voltage curves of perovskite solar cells without C_{60}

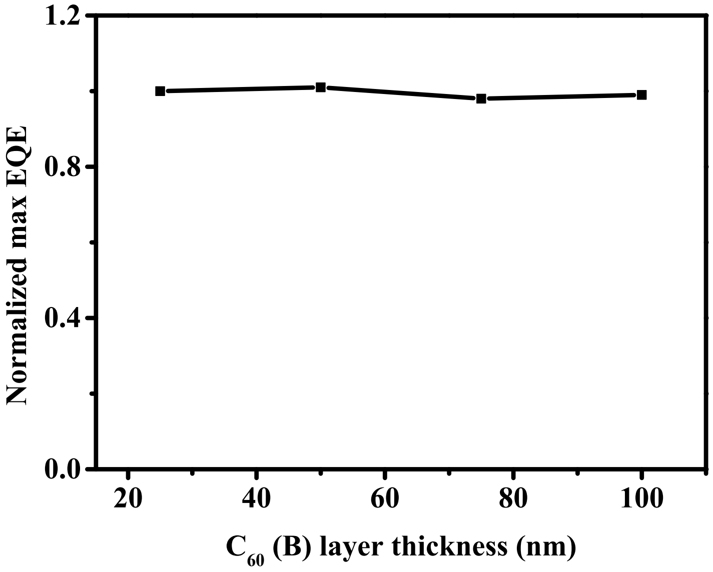

Figure S4 Normalized EQE of PDs with different thicknesses of C_{60} (B) (@ 550 nm and -5V)
